# Questions of Copyright

**DOI:** 10.1186/1477-7525-10-16

**Published:** 2012-01-31

**Authors:** Caroline Anfray, Marie-Pierre Emery, Katrin Conway, Catherine Acquadro

**Affiliations:** 1 MAPI Institute, 27 rue de la Villette, 69003 Lyon, France; 2 MAPI Research Trust, 27 rue de la Villette, 69003 Lyon, France

**Keywords:** Patient-Reported Outcomes, Patient-Reported Outcome Measures, Copyright, Intellectual Property

## Abstract

The Berne Convention and the national laws on intellectual property fully apply to PRO instruments. The identification of and access to an original PRO instrument is often associated with copyright ownership. This is the copyright holder of the instrument who will control its access (distribution and reproduction), its adaptation or modification, and its translation. Copyright is a means to protect the integrity of an instrument. The ownership of an instrument should be defined in the beginning between all parties involved, and each step of the instrument's life, including distribution, should be anticipated for purpose of copyright.

## Correspondence

Recently, the editors of this journal kindly suggested that a brief editorial on the subject of Copyright might be more useful than the comprehensive article on the subject that we had previously submitted. This is our response to their proposal.

We all understand copyright - or do we?

According to the Oxford English Dictionary, copyright is:

"The exclusive legal right, given to an originator or an assignee to print, publish, perform, film, or record literary, artistic, or musical material, and to authorize others to do the same."

No one has a legal right to do *anything *with any original production unless authorized to do so by its originator or an authorized deputy. This includes simple copying, quotation, or manipulation of any kind.

It is worth mentioning at the outset that of a total of some 2,300 requests for information on Patient-Reported Outcomes (PRO) measures received by MAPI Research Trust in 2009, a not-for-profit company, 90% of the questions concerned copyright. The requests were submitted by developers and users of PRO measures as well as publishers.

At various occasion, the Trust has presented a review of the major international instrument on the topic (the Berne Convention for the Protection of Literary and Artistic Works) to help in the exercise of copyright [1-3]. In 2009, Anfray published a discussion about the controversy between Juniper and Grammatopoulou on the 18-item version of the AQLQ(S) [4]. It was at this occasion that Revicki and Schwartz [5] made a clarification of a subject that is more complicated than it first appears. In their editorial, they detail important reasons for developers to exercise their rights. In particular, *"the maintenance of the scientific integrity of the copyrighted instrument which will ensure researchers and readers of scientific journals that the study used the correct version and that there is evidence supporting the psychometric qualities of the instrument." *In the light of the recent FDA guidance on the use of PRO measures, the latter is of special relevance [6].

Questions of copyright may become confused with questions of royalties. Some developers may decide to put their instrument in the public domain to encourage its free and public use. It should be made clear that copyright is not an obstacle to free use if this is the will of the copyright holder. On the other hand, it is perhaps enough to say here that royalties cannot be secured without solving the question of copyright first.

The use of a PRO measure in medical product development is linked to the ability of the user to justify its choice and document its properties. This is clearly stated by regulatory bodies such as the FDA and the EMA, in particular when using an instrument to support a labeling claim [6,7]. To meet these regulatory requirements, and in the light of experience, the following recommendations are offered to developers and users of PROs:

### Recommendations for authors

• Copyright helps to protect the integrity of PRO instruments for scientific purposes.

• Copyright ownership should not be confused with royalty fees. It is not an obstacle to access and use.

• Ownership of copyright in PRO instruments should be established and approved by all parties who participated in the development of the instrument.

• Each step of the instrument's life, including publication and distribution, should be anticipated for the purpose of copyright.

• All agreements should be stated in writing.

• Copyright of a PRO instrument and its derivatives (translations as well as auhtorized modifications) should be the property of the owner of the original instrument, so that there is a single entity centralizing access to all versions.

• Central control of distribution facilitates access to instruments and information about them.

• Developers should register their work in their country of residence as early as possible. *A posteriori *proof of ownership is always difficult.

### Recommendations for users

• The Berne Convention protects *de facto *all PRO measures in the countries adherent to it. Conditions of access must therefore ALWAYS be checked before use.

• License/user agreements should be established in written form.

• The need for additional time for licensing should be anticipated if international trials are involved.

In conclusion, despite its importance for clinical research, the identification of and access to an appropriate original PRO measure and its translations may be challenging and is associated with copyright ownership. Copyright is always situation-specific, and is assured by the existence of written agreements between developers, distributors, and users. It must be clarified before any use.

## Competing interests

The authors declare that they have no competing interests.

## Authors' contributions

CAY and CAC conceived the review of the requests submitted to MAPI Research Trust, participated in its design and coordination, and drafted the manuscript. KC and MPE reviewed the draft papers. All authors read and approved the final manuscript.
